# Targeted Immunotherapy with Rituximab Leads to a Transient Alteration of the IgG Autoantibody Profile in Pemphigus Vulgaris

**DOI:** 10.1155/2010/321950

**Published:** 2010-06-30

**Authors:** Ralf Müller, Nicolas Hunzelmann, Vera Baur, Guido Siebenhaar, Elke Wenzel, Rüdiger Eming, Andrea Niedermeier, Philippe Musette, Pascal Joly, Michael Hertl

**Affiliations:** ^1^Department of Dermatology and Allergology, University of Marburg, D-35033 Marburg, Germany; ^2^Department of Dermatology and Venerology, University of Cologne, D-50937 Cologne, Germany; ^3^Department of Dermatology and Allergology, Ludwig-Maximilian University, D-80337 Munich, Germany; ^4^Department of Dermatology, Rouen University Hospital, F-7600 Rouen, France

## Abstract

In pemphigus vulgaris (PV), IgG autoantibodies against the ectodomain of desmoglein 3 (Dsg3) have been shown to be directly responsible for the loss of keratinocyteadhesion. The aim of the present study was to study the effect of the B cell depleting anti-CD20 monoclonal antibody, rituximab, on the profile of pathogenic IgG against distinct regions of the Dsg3 ectodomain in 22 PV patients who were followed up clinically and serologically by Dsg3 ELISA over 12-24 months. Prior to rituximab, all the 22 PV patients showed IgG against Dsg3 (Dsc3EC1-5). Specifically, 14/22 showed IgG reactivity against the Dsg3EC1 subdomain, 5/22 patients against Dsg3EC2, 7/22 against Dsg3EC3, 11/22 against Dsg3EC4, and 2/22 against Dsg3EC5. Within 6 months after rituximab, all the patients showed significant clinical improvement and reduced IgG against Dsg3 (5/22) and the various subdomains, that is, Dsg3EC1 (7/22), Dsg3EC2 (3/22), Dsg3EC3 (2/22), sg3EC4 (2/22), and Dsg3EC5 (0/22). During the entire observation period, 6/22 PV patients experienced a clinical relapse which was associated with the reappearance of IgG against previously recognized Dsg3 subdomains, particularly against the Dsg3EC1. Thus, in PV, rituximab only temporarily depletes pathogenic B cell responses against distinct subdomains of Dsg3 which reappear upon clinical relapse.

## 1. Introduction

Pemphigus vulgaris (PV) is a life-threatening autoimmune blistering disease caused by IgG autoantibodies (auto-Ab) against the extracellular domain (ECD) of desmoglein 3 (Dsg3) and Dsg1, desmosomal adhesion molecules present on epidermal keratinocytes [1]. Auto-Ab production in PV is polyclonal, and in active PV, most auto-Abs are of the IgG_4_ subclass [2, 3]. It has been postulated that auto-Ab against Dsg3 and Dsg1 primarily target the NH_2_-terminal portion of the Dsg3 ectodomain, that is, the Dsg3EC1 subdomain [4–8]. The EC1 domains of Dsg3 and Dsg1, respectively, according to morphologic studies in desmosomes, are increasingly recognized as the part of the desmosomal cadherin ectodomain which is involved in Dsg transinteraction [9]. Moreover, it seems that IgG auto-Ab reactivity against the Dsg3EC1 domain correlates with active disease in PV although the titers of the auto-Ab do not show a strict correlation with disease activity [10, 11]. Recently, we found a significant correlation between IgG reactivity against Dsg3EC1 and the extent of clinical involvement in PV [8]. IgG against the Dsg3EC1 was seen preferentially in PV patients with mucocutaneous involvement but not in patients with either mucosal or skin involvement only [8]. 

IgG auto-Ab from pemphigus sera cause loss of adhesion of human skin in vivo and in vitro [12, 13]. Because auto-Ab in pemphigus are directed against desmosomal adhesion molecules, it was suggested that these autoantibodies might directly interfere with Dsg transinteraction binding sites [14–17]. Previous studies showed that IgG against the NH_2_-terminus of the Dsg3 ectodomain is pathogenic [18]. Affinity-purified IgG of sera from PV patients injected into neonatal mice which were reactive with the EC1 and EC2 of Dsg3, respectively, induced suprabasilar acantholysis, while IgG reactive with the EC3-5 of Dsg3 did not [10]. AK23, a mouse monoclonal Ab against Dsg3 which targets the predicted binding motif of the Dsg3EC1, has been shown to be pathogenic in vivo whereas IgG targeting other regions of the Dsg3 ectodomain was not [5, 19]. AK23 directly interferes with homophilic adhesion of two Dsg3 proteins [20] which supports the hypothesis that auto-Ab from PV patients directly inhibit Dsg3-mediated epidermal cell adhesion [21]. 

Apart from major advances in our understanding of the immune pathogenesis of pemphigus, therapeutic options in cases of recalcitrant pemphigus are rather limited. The standard immunosuppressive treatment of pemphigus consists of systemic glucocorticoids and adjuvant immunosuppressive drugs which induce partial or complete clinical remission in the majority of the patients. In the remaining refractory cases, the B-cell depleting anti-CD20 monoclonal Ab, rituximab, has been recently introduced as a highly effective rescue medication. Rituximab is a chimeric human/mouse IgG1 monoclonal ab and is directed against CD20, a pan B cell glycoprotein on B lymphocytes from the preB cell to the preplasma-cell stage. Among several mechanisms involved in B cell killing, rituximab exerts B cell cytolytic activity mainly through ab-dependent cell-mediated cytotoxicity. A plethora of case series and two prospective clinical trials strongly suggest that rituximab is highly effective in recalcitrant pemphigus [22].

In the present study, the impact of rituximab treatment on the profile of Dsg3-specific auto-Ab was studied in a cohort of 22 well-characterized patients with PV. During a 12 to 24 months' observation period, IgG reactivity against distinct regions of the Dsg3 ectodomain was correlated with clinical parameters such as involvement of body surface area and mucosal surfaces. Our findings suggest that rituximab treatment only temporarily depletes distinct IgG reactivity against the Dsg3 ectodomain and that the reappearance of such auto-ab is associated with clinical relapses. In particular, IgG reactivity against the NH_2_-terminal EC1 subregion of the Dsg3 ectodomain was preferentially detected in PV patients who experienced a clinical relapse.

## 2. Patients and Methods

### 2.1. PV Patients

Serum samples were obtained from 22 adult patients with PV who were seen at the Dermatology Departments at the Universities of Cologne, Marburg, and Rouen. Patients gave written consent to participate in this study which was adherent to the Declaration of Helsinki Guidelines and which was approved by the local Ethics Committees. The clinical diagnosis of PV was confirmed by (1) histopathology (suprabasal acantholytic blisters), (2) direct immunofluorescence microscopy (epidermal intercellular IgG and/or C3 deposits in perilesional skin), (3) detection of serum IgG auto-ab by indirect immunofluorescence microscopy (intercellular IgG binding to epithelial cells of monkey esophagus), and (4) by ELISA with recombinant Dsg3 and Dsg1. All the 22 PV patients (mean age: 51.3 ± 15.7 years; 11 females and 11 males) mainly presented with mucocutaneous involvement and elevated anti-Dsg IgG titers (Table 1) despite ongoing (> 3 months) immunosuppressive treatment with systemic corticosteroids (initially 1 mg/kg/d; tapered logarithmically upon clinical response) and the immunosuppressive agents, azathioprine (100–150 mg/day) or mycophenolate mofetil (1-2 g/day), respectively (Table 1).

The clinical extent and severity of PV was classified according to the number of blisters or erosions as either mucosal or cutaneous involvement (the later was quantitated by the body surface area; BSA). All the PV patients were treated i.v. with rituximab at 375 mg/m^2^ on days 0, 7, 14, and 21 and were kept on an immunosuppressive treatment regimen consisting of prednisolone and azathioprine or mycophenolate mofetil, respectively.

### 2.2. ELISA with Dsg3 Recombinants

Recombinants representing the entire Dsg3 ectodomain (Dsg3EC1-5) and distinct subregions of the Dsg3 ectodomain, that is, Dsg3EC1-5 (aa 1-566), Dsg3EC1 (aa 1-161), Dsg3EC2 (aa 87-227), Dsg3EC3 (aa 184-349), Dsg3EC4 (aa 313-451) and Dsg3EC5 (aa 424-566) were produced in an eukaryotic baculovirus expression system and were purified using Nickel-NTA as previously described [8, 23]. By ELISA, the patients' sera were tested for IgG reactivity against the Dsg3 ectodomain (Dsg3EC1-5) as well as IgG reactivity against subdomains of the extracellular portion of Dsg3 as recently described [8, 23]. In short, the recombinant Dsg proteins were immobilized on microtiter plates and were incubated with the PV patients' sera at a dilution of 1 : 50. IgG binding was visualized as optical density (OD) at 405 nm. Samples were run at least in duplicate and OD's were expressed as mean values. The threshold for IgG binding of the Dsg3 recombinants in the ELISA yielded an OD value of 0.376 as determined by a ROC curve generated for Dsg3EC1-5 [23]. OD values above the cutoff point defined positive IgG reactivity, while IgG reactivity below the cutoff point was considered to be negative.

### 2.3. Scoring of Disease Activity in Pemphigus

Scores for mucosal involvement were set as follows: 0, if the patient had no mucosal involvement, 1 point for each additional location of blisters (i.e., oral, dysphagia, genital, conjunctival involvement) and additionally, 1 point for >3 erosions with a diameter >2 mm. Skin involvement was assessed by body surface area and was expressed as percentage of affected area.

## 3. Results

### 3.1. Clinical Response to Rituximab of the Studied PV Patients

**Figure 1:**
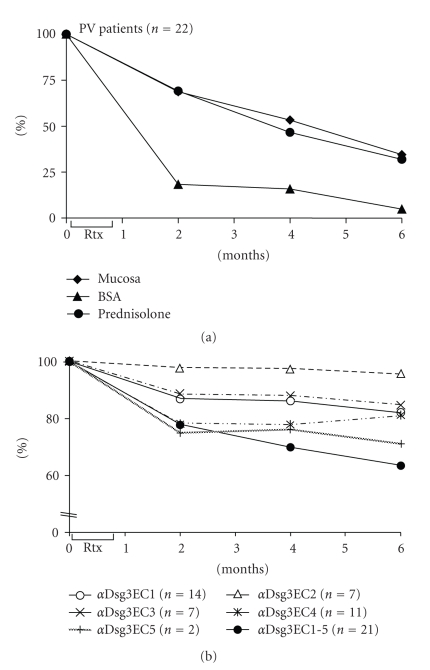
Rituximab (Rtx) treatment of pemphigus vulgaris (PV) patients leads to clinical improvement of skin and mucosal lesions and is accompanied by decreased IgG titers against distinct regions of desmoglein 3 (Dsg3) ectodomain. Treatment with Rtx of 22 PV patients led to a significant improvement of mucosal and skin lesions and is expressed as reduced mucosal and body surface area (BSA) scores within 6 months after treatment (Figure 1(a)). In the same way, the dose of prednisolone was reduced (Figure 1(a)). By enzyme-linked immunosorbent assay, there was clearly a reduction of IgG reactivity against the Dsg3 ectodomain (Dsg3EC1-5) and defined regions of the Dsg3 ectodomain (Figure 1(b)). The number of studied patients or sera, respectively, is given in parenthesis. Rtx (time when rituximab was administered).

All the 22 PV patients showed a marked clinical response to rituximab treatment which was expressed as dramatically reduced mucosal (baseline: 4.60 ± 1.96; 1.59 ± 1.80 after 6 months) and BSA (baseline: 11.05 ± 13.14; 0.53 ± 1.01 after 6 months) scores within six months after treatment (Figure 1(a)). Among the studied patients, 18/22 (81.8%) had skin involvement and 18/22 (81.8%) mucosal involvement. Except for patient PV6 whose clinical symptoms were unknown, all the PV patients had mucosal involvement. Clinical improvement to rituximab was also monitored as a marked reduction of systemic treatment with prednisolone from 0.63 ± 0.60 mg/kg body weight (baseline) to 0.20 ± 0.17 mg/kg body weight (6 months after rituximab treatment) (Figure 1(a)). Noteworthy, 6/22 PV patients experienced a relapse 12 to 24 months after rituximab treatment.

### 3.2. IgG Titers against Distinct Dsg3 Subdomains and Disease Activity of the PV Patients Are Reduced upon Treatment with Rituximab

IgG auto-ab of the 22 PV patients against the Dsg3 ectodomain and its subdomains was analysed by ELISA during the course of the disease (Figure 1(b)). Prior to treatment with rituximab, all PV patients showed IgG reactivity against at least some part of the entire Dsg3 ectodomain (Dsg3EC1-5), 14/22 showed IgG reactivity against the Dsg3EC1 subdomain, 5/22 patients against Dsg3EC2, 7/22 against Dsg3EC3, 11/22 against Dsg3EC4, and 2/22 against Dsg3EC5. Within 6 months after treatment, there was a marked reduction of IgG reactivity against the Dsg3 ectodomain as well as against distinct subdomains (Figure 1(b)). Because of the availability of patients sera at all time points, the persistence or disappearance of IgG against distinct Dsg3 regions upon treatment with rituximab was studied in more detail in 16 PV patients. 

**Figure 2:**
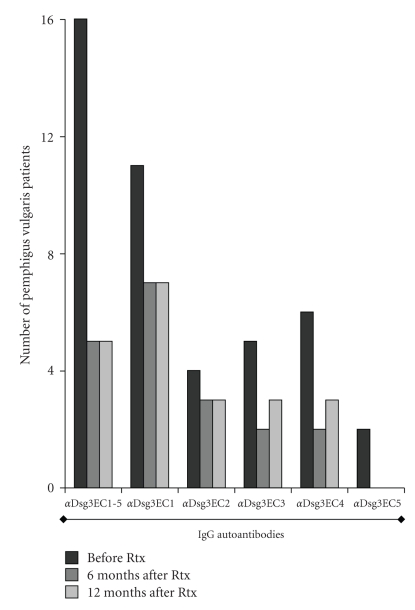
IgG reactivity against desmoglein 3 (Dsg3) subdomains in pemphigus vulgaris (PV) patients on rituximab (Rtx). Illustrated is the number of PV patients showing IgG against Dsg3 subdomains before and 6 and 12 months after treatment with Rtx, respectively.

Among these patients, the number of patients with IgG reactivity against the recombinant proteins of Dsg3 of less than 80% of the initial level was studied 6 and 12 months after rituximab (Figure 2). Initially, 16 PV patients showed IgG reactivity against Dsg3EC1-5, 11 against Dsg3EC1, 4 against Dsg3EC2, 5 against Dsg3EC3, 6 against Dsg3EC4, and 2 against Dsg3EC5. Of the 16 patients, a total of 11 showed reduced (by more than 80%) IgG reactivity against the Dsg3 ectodomain (Dsg3EC1-5): Six months after treatment with rituximab, IgG reactivity against Dsg3EC1 was more than 80% reduced in 4/11 patients, against Dsg3EC2 in 1/4 PV patients, against Dsg3EC3 in 3/5 patients against Dsg3EC4 in 4/6, and against Dsg3EC5 in 2/5 patients. Twelve months after treatment with rituximab, IgG reactivity of less than 80% of the initial value was noticed in 11/16 (Dsg3EC1-5), 4/11 (Dsg3EC1), 1/4 (Dsg3EC2), 2/5 (Dsg3EC3), 3/6 (Dsg3EC4) and 2/2 (Dsg3EC5) PV patients. Specifically, the impact of rituximab treatment on IgG reactivity against distinct Dsg3 subdomains is given in Table 2 (0 and 6 months).

### 3.3. Reappearance of IgG against Distinct Regions of the Dsg3 Ectodomain in PV Patients with Clinical Relapses after Rituximab Treatment

**Figure 3:**
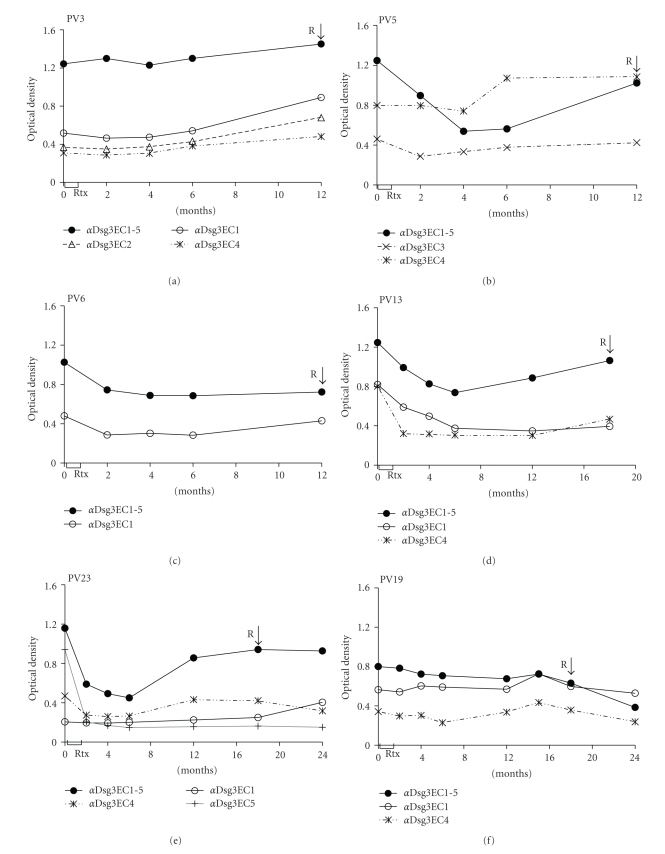
Association of clinical relapses of rituximab (Rtx)-treated pemphigus vulgaris (PV) patients and the reappearance of IgG against distinct regions of the Dsg3 ectodomain. Three of the 6 PV patients (PV3, PV6, PV23) showed an increase of IgG reactive with the Dsg3EC1 subdomain upon clinical relapse after Rtx therapy. Patient PV13 showed increased serum levels of IgG against the Dsg3EC4 subdomain while patients PV5 and PV19 did not show altered IgG levels against any of the studied Dsg3 subdomains upon clinical relapse.

Six of the 22 PV patients treated with rituximab showed a clinical relapse 12 months (*n* = 3; PV3, PV5, PV6) or 18 months (*n* = 3; PV13, PV19, PV23), respectively, after rituximab treatment (Table 3). Clinical relapses were defined as newly arisen mucosal or cutaneous lesions which persisted for more than 7 days. All the relapsed PV patients showed increased IgG auto-ab against at least some part of the entire Dsg3 ectodomain (Figure 3) but with individual patterns of IgG recognition of Dsg3 subdomains (Table 3). In the individual PV patients, clinical relapses were associated with the reappearance of distinct auto-ab profiles (Figure 3). Four of the 6 relapsed PV patients had IgG against the Dsg3EC1 subdomain prior to rituximab treatment, that is, patients PV3, PV6, PV13, and PV19 (Figure 3). In these PV patients, IgG auto-ab against Dsg3EC1 decreased (PV6, PV13) or remained constant (P3, P19) upon rituximab treatment. Noteworthy, in all these patients, IgG against the Dsg3EC1 persisted until or reappeared at the time of clinical relapse. PV patient PV23 did not initially show IgG against Dsg3EC1 but at the time of relapse 18 months after rituximab treatment. 

Only PV patient PV5 had a different auto-ab profile and showed IgG against Dsg3EC3 and Dsg3EC4 prior to rituximab. At the time of clinical relapse 12 months later, he showed IgG against Dsg3EC4 which exceeded pretreatment levels (Figure 3). In patient PV5, IgG against Dsg3EC1 was not detected at the time of clinical relapse. 

 Noteworthy, some of these patients showed IgG reactivity against distinct Dsg3 subdomains in addition to IgG against Dsg3EC1 at the time of clinical relapse. Patient PV3 showed IgG against Dsg3EC1, Dsg3EC2, and Dsg3EC4 when he developed new cutaneous lesions and patient PV5 had IgG against Dsg3EC1, Dsg3EC2, Dsg3EC3, and Dsg3EC4 when he relapsed. Finally, PV patient PV23 showed IgG against Dsg3EC1 and Dsg3EC4 upon clinical relapse. None of the 6 PV patients showed IgG against the COOH-terminal Dsg3EC5 subdomain at the time of clinical relapse (Figure 3). 

## 4. Discussion

The present study strongly suggests that rituximab, a monoclonal antibody against CD20 on B cells, does not permanently deplete pathogenic B cell responses against Dsg3 in patients with PV, a potentially lethal autoimmune bullous disorder of skin and mucosa. Here, a total of 22 patients with refractory PV were treated with rituximab which had been recently identified as a potent treatment in refractory PV [24, 25]. Specifically, these patients did not adequately respond to standard immunosuppressive treatment consisting of high dose systemic corticosteroids combined with the immunosuppressive adjuvants, azathioprine, or mycophenolate mofetil, respectively. 

In the cohort of studied patients, all of the 22 PV patients initially showed an excellent clinical response to rituximab treatment. However, within 12–18 months after rituximab treatment, 6/22 PV patients experienced a clinical relapse which was associated with the persistence or reappearance of IgG auto-ab against distinct extracellular subdomains of Dsg3, the major autoantigen of PV (Figure 3). The majority, that is, 5/6 of the relapsed PV patients, showed the reappearance of IgG against the NH_2_-terminal subdomain Dsg3EC1 which has been previously identified as the site where most of the pathogenic PV auto-Ab bind [11, 19, 26, 27]. In addition, several of the relapsed patients showed IgG reactivity against additional subdomains of Dsg3 most of which had initially disappeared upon treatment with rituximab. Of note, IgG reactivity against the COOH-terminal EC5 subdomain of Dsg3 was not observed in relapsed patients. This region which is located close to the keratinocyte cell membrane is presumably not a target for pathogenic auto-ab in PV [11]. Thus, IgG reactivity against distinct regions of the Dsg3 subdomain which was abolished by rituximab treatment reappeared in these patients at the time of clinical relapse strongly suggesting that the repertoire of Dsg3-specific B cells was only temporarily deleted by rituximab treatment. The frequent detection of Dsg3EC1-specific IgG in clinically relapsed PV patients supports the concept that pathogenic PV auto-ab preferentially target the NH2-terminal EC1 domain of Dsg3. The relative pathogenic role of IgG against Dsg3 subdomains other than Dsg3EC1 awaits further analysis.

The majority, that is, 16/22 PV patients, were successfully controlled by treatment with rituximab and prednisolone over 12 and 24 months, respectively. The other 6 patients showed a clinical relapse during the observation period. Our findings with rituximab are in line with previous reports demonstrating an excellent clinical response associated with a strong reduction of Dsg1- and Dsg3-specific IgG levels on adjuvant treatment with rituximab [2, 25].

Of note, clinical improvement of mucosal and skin lesions of the rituximab-treated PV patients was significantly associated with a decrease of IgG auto-ab titers. However, we did not find any significant correlation between IgG against distinct subdomains of Dsg3 and skin or mucosal involvement. However, our findings suggest that IgG against the Dsg3EC1 domain is associated with active PV as shown earlier by our group and others [5, 8, 11, 23]. The Dsg3 recombinants of the present studies may not contain all the conformational epitopes that are relevant in the immune pathogenesis of pemphigus. However, as proven in two recent reports, these findings with these Dsg3 recombinants are valid and show differential reactivity against distinct portions of the Dsg3 ectodomain in different pemphigus patients and different diseases stages [8, 23]. 

Recently, we found a significant correlation between IgG reactivity against the Dsg3EC1 and the extent of clinical involvement in PV [8]. IgG against the Dsg3EC1 was seen preferentially in PV patients with muco-cutaneous involvement but not in patients with either mucosal or skin involvement only [8]. IgG auto-ab from pemphigus sera are sufficient to cause blistering in human skin in vivo and in vitro [12, 13] by direct interference with Dsg transinteraction binding sites [14–17]. 

Previous studies showed that IgG against the NH_2_-terminus of the Dsg3 ectodomain is pathogenic [18]. Affinity-purified IgG from sera of PV patients injected into neonatal mice which were reactive with the EC1 and EC2 of Dsg3 induced suprabasilar acantholysis, while IgG reactive with the EC3-5 of Dsg3 did not [10]. AK23, a mouse monoclonal ab against Dsg3 which targets the predicted binding motif of the Dsg3EC1, has been shown to be pathogenic in vivo whereas IgG that targets other parts of the Dsg3 ectodomain was not [5, 19]. Additionally, AK23, is able to directly interfere with homophilic Dsg3 binding [20] which supports the hypothesis that auto-ab from PV patients directly inhibit Dsg3-mediated epidermal cell adhesion [21]. 

Sekiguchi et al. suggested that, both pathogenic and nonpathogenic auto-ab exist in PV [5]. This heterogeneity among anti-Dsg3 antibody due to specific epitope recognition was previously demonstrated [19]. In addition to pathogenic IgG auto-ab against Dsg3EC1, IgG against additional Dsg3 subdomains, that is, DsgEC2, DsgEC3, and Dsg3 EC4, may not be pathogenic per se but may act synergistically in inducing the pathology of intraepidermal loss of adhesion [8]. This contention is supported by a recent study showing that nonpathogenic monoclonal IgG ab that recognize regions of the Dsg3 ectodomain other than the NH_2_-terminus induce desmosomal loss of adhesion when injected together into mice [28]. 

 Further studies are necessary to investigate the dynamics of pathogenic IgG against the Dsg3EC1 and auto-ab against other Dsg3 subdomains in the immune pathogenesis of PV. At present, it remains to be elucidated why rituximab only temporarily depletes pathogenic auto-ab in PV. Previous observations from our laboratory strongly suggest the presence of short-lived, Dsg3-reactive plasma cells since rituximab rapidly reduced Dsg3-specific IgG auto-ab but not IgG against recall antigens such as tetanus toxoid, Epstein Barr virus or influenza virus [29, 30]. Based on the present and previous observations, detection and monitoring of IgG against the Dsg3EC1 may be a more sensitive marker of disease activity in patients with PV than monitoring IgG against the entire Dsg3 ectodomain.

## Figures and Tables

**Table 1:** Clinical and immunological characteristics of the pemphigus vulgaris (PV) patients on adjuvant treatment with rituximab.

Patients	Treatment^(1)^	Sex	Age	Clinical phenotype of PV^(2)^	Severity^(3)^		IgG autoantibody profile^(4)^	
					Mucosa	BSA (%)	Anti-Dsg1	Anti-Dsg3
PV1	Pred., MMF, Dapson	m	53	mucocutaneous	2	3	103	549
PV2	Pred., MMF, MTX	m	67	mucocutaneous	4	1	10	484
PV3	Pred., MMF, Azathioprin	f	69	mucosal	7	0	neg	810
PV4	Pred.	f	69	no mucosal involvement	0	4	163	2368
PV5	Pred. MMF, Azathioprin	f	37	mucocutaneous	3	5	183	94
PV6	n.d.	m	48	n.d.	n.d.	n.d.	979	906
PV7	Pred., MMF	m	50	mucocutaneous	8	1	82	201
PV8	Pred., MMF, Dapson	f	23	mucocutaneous	2	12	357	145
PV10	Pred., MMF	f	57	mucosal	6	0	3	124
PV11	Pred., MMF, MTX	f	28	mucosal	5	0	6	229
PV12	Pred., MMF	f	54	mucocutaneous	7	5	199	175
PV13	n.d.	m	62	mucocutaneous	7	10	78	220
PV14	Pred.	m	79	mucocutaneous	6	10	170	200
PV15	Pred.	m	36	mucocutaneous	4	60	155	201
PV16	n.d.	m	64	mucocutaneous	6	20	163	58
PV17	Pred.	f	51	mucocutaneous	5	10	181	209
PV18	Pred.	f	70	mucocutaneous	4	10	neg	143
PV19	n.d.	m	59	mucocutaneous	4	10	98	156
PV20	Pred.	m	38	mucocutaneous	4	20	36	138
PV21	Pred.	f	41	mucocutaneous	5	10	18	119
PV22	Pred.	m	24	mucocutaneous	4	20	138	135
PV23	Pred.	f	50	Mucocutaneous	5	10	176	163

^(1)^Systemic prednisolone (Pred) was administered throughout the observation period and was logarithmically tapered upon clinical reponse to treatment; azathioprine (AZA) or mycophenolate mofetil (MMF), respectively, was also administered throughout the observation period. n.d., not determined.

^(2)^Before treatment with rituximab.

^(3)^Determined by the extent of cutaneous involvement as body surface area (BSA) and the extent of mucosal involvement.

^(4)^Determined by enzyme-linked immunosorbent assay (ELISA) with recombinant desmoglein (Dsg) 1 and 3; optical densities are expressed as PIV (protein index value).

^(5)^PIV cutoff is 20.

**Table 2:** Profile of desmoglein 3 (Dsg3)-specific IgG autoantibodies of the pemphigus vulgaris (PV) patients before and 6 months after treatment with rituximab (Rtx).

	Dsg3EC1		Dsg3EC2		Dsg3EC3		Dsg3EC4		Dsg3EC5	
Time point	0	6	0	6	0	6	0	6	0	6
PV1	+	+	+	+	+	+	−	−	−	−
PV2	+	+	+	+	−	−	−	−	−	−
PV3	+	+	−	+	−	−	−	+	−	−
PV4	+	−	−	−	+	+	+	−	−	−
PV5	−	−	−	−	+	−	+	+	−	−
PV6	+	−	−	−	−	−	−	−	−	−
PV7	+	+	+	+	−	−	−	−	−	−
PV8	−	−	−	−	+	−	−	−	−	−
PV10	−	−	−	+	−	−	+	−	−	−
PV11	+	+	−	−	−	−	−	−	−	−
PV12	+	+	+	−	+	+	+	+	−	−
PV13	+	−	−	−	−	−	+	−	−	−
PV14	+	−	+	−	−	−	+	−	+	−
PV15	−	−	−	−	+	−	−	−	−	−
PV16	−	−	−	−	−	−	+	+	−	−
PV17	+	+	−	−	−	−	−	−	−	−
PV18	−	−	−	−	−	−	+	−	−	−
PV19	+	+	−	−	−	−	−	−	−	−
PV20	+	+	−	−	−	−	−	−	−	−
PV21	+	+	−	−	+	+	+	−	−	−
PV22	−	−	−	−	−	−	+	−	−	−
PV23	−	−	−	−	−	−	+	+	+	−

**Table 3:** Synopsis of the IgG autoantibody profile of the pemphigus vulgaris (PV) patients prior to rituximab (preRtx) treatment and at the time of clinical relapse (CR).

IgG against extracellular subdomains of Dsg3															
	Dsg3EC1			Dsg3EC2			Dsg3EC3			Dsg3EC4			Dsg3EC5		
Time point	preRtx	6 months	CR	preRtx	6 months	CR	preRtx	6 months	CR	preRtx	6 months	CR	preRtx	6 months	CR
PV3 (CR, months 12)	+	+	+	−	+	+	−	−	−	−	+	+	−	−	−
PV5 (CR, months 12)	−	−	−	−	−	+	+	−	+	+	+	+	−	−	−
PV6 (CR, months 12)	+	−	+	−	−	−	−	−	−	−	−	−	−	−	−
PV13 (CR, months 18)	+	−	+	−	−	−	−	−	−	+	−	+	−	−	−
PV19 (CR, months 18)	+	+	+	−	−	−	−	−	−	−	−	+	−	−	−
PV23 (CR, months 18)	−	−	+	−	−	−	−	−	−	+	+	+	+	−	−

## Abbreviations

Autoantibody: auto-ab
Dsg: desmoglein
Dsg3EC1-5: baculoprotein representing the entire ectodomain (aa1-566) of desmoglein 3
Dsg3EC1: baculoprotein containing aa1-161 of desmoglein 3
Dsg3EC2: baculoprotein containing aa87-227 of desmoglein 3
Dsg3EC3: baculoprotein containing aa184-349 of desmoglein 3
Dsg3EC4: baculoprotein containing aa313-451 of desmoglein 3
Dsg3EC5: baculoprotein containing aa424-566 of desmoglein 3
PV: pemphigus vulgaris.
